# Older Adults’ Acceptance of a Virtual Reality Group Intervention in Nursing Homes: Pre-Post Study Under Naturalistic Conditions

**DOI:** 10.2196/56278

**Published:** 2024-08-06

**Authors:** Yijun Li, Irina Shiyanov, Beate Muschalla

**Affiliations:** 1 Department of Psychotherapy and Diagnostics, Institute of Psychology, Technische Universität Braunschweig, Humboldtstraße 33, Braunschweig, 38106, Germany, 49 0531 391-3603; 2 VirtuaLounge GmbH, Braunschweig, Germany

**Keywords:** virtual reality, VR, computer-generated simulation, simulation, technology acceptance, nursing home, nursing facility, long-term care center, long-term care facility, older adult, elder, elderly, older person, older people, senior, understanding human behavior, meaningful activity, group intervention, human behavior

## Abstract

**Background:**

Virtual reality (VR) group activities can act as interventions against inactivity and lack of meaningful activities in nursing homes. The acceptance of VR among older adults has been explored from different perspectives. However, research on the impact of older adults’ individual characteristics on the acceptance of VR group activities in nursing homes is necessary.

**Objective:**

This study investigates the impact of individual characteristics (eg, psychosocial capacities) on VR acceptance among older adults in nursing homes, as well as this group’s perceptions of VR after participating in a VR intervention.

**Methods:**

In this pre-post study conducted in nursing homes, we applied a VR group intervention with 113 older adult participants. These participants were categorized into two groups based on their naturalistic choice to join the intervention: a higher VR acceptance group (n=90) and a lower VR acceptance group (n=23). We compared the two groups with respect to their sociodemographic characteristics, psychosocial capacities, and attitudes toward new technologies. Additionally, we examined the participants’ perceptions of VR.

**Results:**

The results show that those with lower acceptance of VR initially reported higher capacities in organizing daily activities and stronger interpersonal relationships compared to older adults with higher VR acceptance. The VR group activity might hold limited significance for the latter group, but it offers the chance to activate older adults with lower proactivity. Openness to new technology was associated with a favorable perception of VR. After the VR intervention, the acceptance of VR remained high.

**Conclusions:**

This study investigates the acceptance of VR group events as meaningful activities for older adults in nursing homes under naturalistic conditions. The results indicate that the VR group intervention effectively addressed low proactivity and interpersonal relationship issues among older adults in nursing homes. Older adults should be encouraged to experience VR if the opportunity to participate is offered, potentially facilitated by caregivers or trusted individuals.

## Introduction

The absence of meaningful activities and a lack of social interaction can lead to loneliness in older adults in nursing homes [1,2]. Meaningful activities are defined as those that hold personal significance or offer enjoyment to individuals, aligning with their present and past interests, routines, habits, and roles [3,4]. In the context of nursing homes, a meaningful activity could include household tasks like cooking, which older adults may no longer be able to do on their own. A lack of meaningful activity leads to older adults’ inactivity, resulting in mental and physical impairment, as well as an increased risk of mortality [5-8]. Furthermore, several studies show that older adults in nursing homes experience more loneliness compared to those living in the community, even though they are often surrounded by other residents and caregivers [9,10]. Addressing these issues requires innovative solutions [8,11,12]. Virtual reality (VR) has emerged as a promising solution in rehabilitation [13,14], including for older adults [1,15,16]. VR technology, especially fully immersive VR technology, delivers a comprehensive and lifelike experience, creating a strong sense of presence for users by using head-mounted displays and motion-tracking controllers. These devices work in tandem to simulate a realistic, interactive environment, allowing users to see, hear, and interact with the virtual world in a manner similar to real-world experiences [17]. Various studies have shown that VR interventions can enhance cognitive capacities [18,19], improve physical strength [16,18,20]—for example, via walking training [21]—and enhance the overall well-being of older adults [16,19,21-23]. Moreover, the immersive nature of VR not only conserves resources and reduces costs but also ensures safety [24-26]. Thus, VR can be a viable choice for providing meaningful activities within nursing homes to enhance the daily activities, capacities, social activities, and well-being of older adults in nursing homes [27].

In a study on the acceptance of VR technology that included 76 older adults, it was found that participants developed a positive attitude toward VR after using it [17]. A systematic review and meta-analysis found that in most of the 54 relevant studies, older adults reported pleasant experiences with VR and expressed a desire to use it again [28]. In addition to assessing the level of acceptance of VR among older adults, it is also necessary to understand the factors that could influence this acceptance [29]. According to the Technology Acceptance Model (TAM) [30], the acceptance of a technology can be explained and positively predicted by perceived usefulness and perceived ease of use. In the context of our study, the implementation of VR technology should address specific needs, such as providing meaningful activities and combating loneliness, and the design of the VR intervention should be in alignment with the capacities of nursing home residents to ensure the perceived ease of use of the intervention. However, this requires an in-depth understanding of the characteristics of the target population. The field of gerontechnology has advanced in developing frameworks to address the unique capacities and limitations of older adults. A notable gap in the previous acceptance models [31] is the insufficient exploration of personal characteristics. In addition to collecting demographic factors, research should explore participants’ cognitive capacities, social relationships, environmental influences, and psychosocial traits. Previous research about VR acceptance among older adults has similarly highlighted the importance of individual characteristics such as physical constraints, educational attainment, and socioeconomic status as predictive factors [32,33]. A qualitative analysis about the acceptance of technology among older adults [34] found noticeable differences in attitude linked to participants’ educational background and work experiences, but no definitive differences were found with regard to gender and ages. Older age negatively impacted the willingness of participants to use robots [35]. Further studies are needed to confirm the acceptability of different types of immersive technology devices [28], including a detailed report on VR interventions [29]. An in-depth investigation into personal characteristics, including how the sociodemographic factors and psychosocial capabilities of nursing home residents affect their acceptance of VR group activities, will be valuable for understanding these dynamics and contributing to future design improvements.

Besides sociodemographic characteristics, technological engagement is another important factor for individual technological acceptance. Empirical findings underscore the pivotal role of openness to new experiences as a predictor of improved digital acceptance [36]. Individuals with a higher motivation for self-actualization tend to be more receptive to new encounters and the acquisition of novel skills and ideas [37]. This propensity for continuous learning aligns with the potential for individuals to actualize their personal capacities through the acquisition of new technological skills [38]. In addition, prior knowledge about VR has been identified as a decisive factor in VR acceptance [32]. This finding resonates with the continuity theory, which posits that older adults make adaptive choices to maintain ties with their past experiences [39]. Adhering to established habits rooted in past experiences serves to mitigate the uncertainty that may accompany new environments. This preference for continuity extends to the perpetuation of personal cognitive frameworks shaped by past preferences. Attitudes toward immersive VR have been observed to change from neutral to positive after the first exposure [17]. Therefore, older adults with higher technical engagement prior to the study may have a higher acceptance of VR-based group activities.

Anxiety toward new technologies, which significantly influences technology acceptance, is rooted in factors like unfamiliarity with computers, perceived uncertainty, fear, and a general apprehension toward making mistakes [40,41]. There is a significant correlation between elevated levels of self-efficacy and reduced anxiety with increased utilization of gerontechnology [42]. In essence, this implies that for older adults to embrace novel technology, they must overcome the fear of uncertainty by taking the initial step of trying it out, preferably with sufficient support. Users tend to experience contentment with new technology once they start using it [43-45]. Therefore, after participating in a VR intervention, the acceptance of new technologies, especially VR technology and VR group events, should be maintained or even improved.

This study focuses especially on the impact of individual characteristics of older adults on the acceptance of VR-based group activities in nursing homes. A series of VR interventions was conducted to address the residents’ lack of meaningful activities and loneliness. Over the course of 4 VR intervention sessions, older adults engaged in serious games involving tasks that they may no longer be able to perform within the nursing home environment, such as cooking and gardening. This aligns with the concept of meaningful activities. This study aimed to investigate whether acceptance of VR is linked to personal characteristics, such as sociodemographic background, psychosocial capacities for nursing home residency, and technological engagement. Therefore, the research question and hypotheses of this paper are the following:

- Research question: What are the differences in sociodemographic status and psychosocial capacities between older adults with higher and lower acceptance of using a VR intervention for meaningful activity in nursing homes under naturalistic conditions?
- Hypothesis 1: The group exhibiting higher acceptance of a VR group activity reports a higher level of technological engagement compared to the group with lower VR acceptance.
- Hypothesis 2: After the VR group intervention, technological engagement among older adults will either be maintained or improved.
- Hypothesis 3: After the VR group intervention, the acceptance of VR technology and willingness to participate will both align at a high level.

Our research addresses critical gaps in the existing literature. First, our investigation focused on a specific population and implementation of VR technology: older adults living in nursing homes and VR group events as meaningful activities, respectively. Second, the study focused on differences in personal capacities and was not limited to demographic background. Third, the study was done under naturalistic conditions with high ecological validity, that is, using VR in a group event in a nursing home and collecting naturalistic data. This investigation holds the potential to provide a more nuanced classification and predictive understanding of the specific demographics within nursing homes. It can pave the way for the future development of VR programs that are precisely tailored to the individual needs and preferences of older adults in nursing homes.

## Methods

### Research Design and Recruiting

We conducted a VR group intervention study as a pre-post observation study in naturalistic settings with older adults in 14 nursing homes in a city of 250,000 inhabitants in Germany.

In a first step, the nursing homes were contacted via telephone. After receiving confirmation of willingness to participate from the institution, an email with the participant selection criteria was sent to the social coordinator of each nursing home. The older adults were selected by the nursing home caregivers based on the following criteria: (1) the participant should be older than 60 years; (2) they can use at least one arm and hand for interacting with the system; (3) they can see and hear, with assistive devices such as glasses allowed; (4) their cognitive and mental capacity are sufficient for individual interviews; (5) they do not have conditions that could be triggered by VR, such as epilepsy. In addition to meeting these criteria, caregivers asked the older adults about their willingness to participate in the VR intervention. Furthermore, if needed, permission was sought from legal guardians.

### Procedure

**Figure 1.**
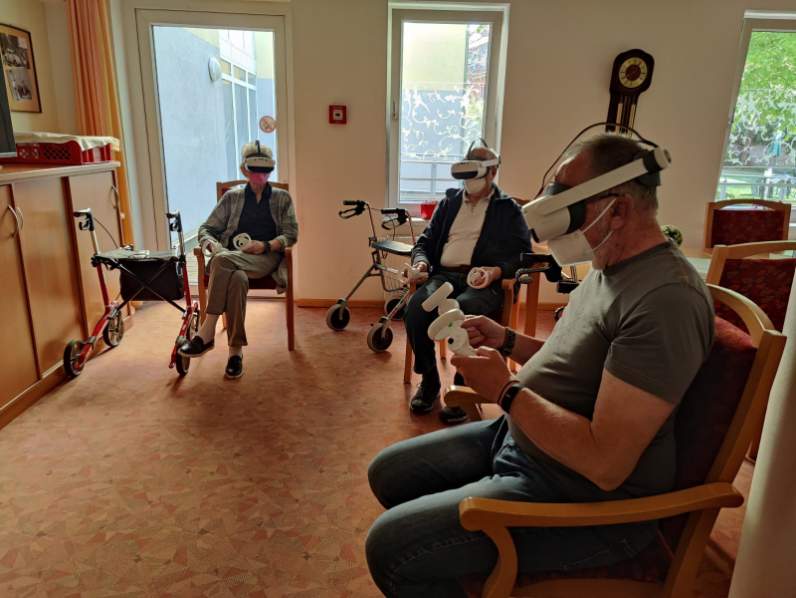
Older adults participating in a virtual reality group event.

In the initial study week, a structured individual interview was conducted with the older adults by a psychologist. Baseline sociodemographic characteristics, capacities, and technological inclination of the older adults were assessed (T1). Following a warm-up interview in the second week (T2), the subsequent 4 weeks included repeated exposure to VR group activities (T3-T6). During these sessions, the older adults were organized into groups, with each group consisting of a maximum of 5 members. These groups remained consistent throughout the entire VR intervention. The older adults were expected to accomplish designated tasks individually (Figure 1). After the VR intervention, participants exchanged thoughts about their experiences in small groups. Moreover, an immersive VR video was provided for relaxing after each intervention. After completion of all VR interventions, a posttest was used to assess the older adults’ technological engagement (T7).

Participants declining VR intervention involvement were offered participation in a control group that underwent an identical measurement and interview procedure excluding the VR intervention (Table 1).

**Table 1.** Procedure of the VR^a^ group intervention study in nursing homes.

	Week 1	Week 2	Weeks 3‐6	Week 7	Week 10
Event	Baseline (T0)	Warm-up (T1)	VR interventions (T2-T5)	Posttest (T6)	Follow-up (T7)
Content	Interview: Demographic information Mini-ICF-APP Technological engagement Perception of VR	Short interview	VR interventions	Interview: Technological engagement Perception of VR	Interview

a VR: virtual reality.

### Participants

To answer our research questions, we needed to compare participants with higher and lower acceptance of the VR intervention. The necessary sample size for group comparison with a *t* test for independent groups, with a medium effect size *d*=0.5, an α level of .05, and a power of 1–β=0.80, was calculated to be 102 participants, with 51 participants in each group.

We collected data from 129 participants, of which 113 were relevant to this analysis. The composition of the groups, based on the naturalistic characteristics of the participants (higher or lower VR intervention acceptance), was unpredictable, resulting in an unequal group size.

Initially, a total of 129 older adults in nursing homes participated in the VR intervention study, of which 12 opted for the control group and 117 opted for the intervention group. Among these participants, 27 individuals in the intervention group discontinued their involvement in the VR intervention. The reasons for dropout and nonparticipation in the intervention group were categorized as non–motivation-related (eg, health, life status; n=16) or motivation-related (n=11). Non–motivation-related reasons included illness (switch to hospital stay), limited station participation (no group established), and cybersickness. The non–motivation-related dropouts were not investigated further in this study. Motivation-related reasons included diminished interest after the initial interview and having a preference for an alternate activity. In the end, there were 90 older adults in the intervention group who completed the posttest.

The sample that was analyzed to answer our research questions included 113 participants, that is, the initial sample (n=129) reduced by the participants who dropped out due to nonmotivational reasons (n=16). The investigated sample was categorized into two groups: individuals with higher or lower acceptance of the VR intervention. The higher acceptance group encompassed the participants (n=90) who actively participated in the VR interventions and successfully completed the subsequent postinterview. The lower acceptance group (n=23) comprised those who dropped out of the intervention group due to motivation-based reasons and older adults who initially were not interested (control group). The higher VR acceptance group was younger (mean 80.13, SD 8.39 years) than the lower VR acceptance group (mean 83.74, SD 8.31 years), but this was not significant (*t*_111_=–1.84; *P*=.07; *d*=–.43). For comprehensive sociodemographic details of these two groups, refer to Table 2.

**Table 2.** Sociodemographic data of older adults who participated in the VR^a^ intervention.

		Higher VR acceptance^b^ (n=90), n (%)	Lower VR acceptance^c^ (n=23), n (%)	Chi-square (*df*)	*P* value
Sex (female)		59 (65.6)	18 (78.3)	1.36 (1)	.24
**Education**				8.61 (5)	.13
	None	6 (6.7)	3 (13)		
	Primary school	1 (1.1)	0 (0)		
	Lower secondary school (ninth or tenth grade)	57 (63.3)	11 (47.8)		
	Upper secondary school	16 (17.7)	9 (39.1)		
	A-levels	10 (11.1)	0 (0)		
**Professional qualification**				1.61 (3)	.68
	None	27 (30)	7 (30.4)		
	Apprenticeship or skilled work	52 (57.8)	15 (65.2)		
	Master craftsman	6 (6.7)	1 (4.3)		
	University studies	5 (5.6)	0 (0)		
**Longest professional activity in working life**				3.04 (6)	.80
	Crafts, industry, production	30 (33.3)	6 (27.3)		
	Research and development	3 (3.3)	0 (0)		
	Agriculture	1 (1.1)	1 (4.5)		
	Office, management	21 (23.3)	5 (22.7)		
	Service, gastronomy, customer service	20 (22.2)	1 (4.5)		
	Practical health care (eg, nurse, doctor, therapist)	7 (7.8)	1 (4.5)		
	Housewife	8 (8.9)	2 (9.1)		
**Frequency of visits from trusted people**				4.85 (5)	.43
	Several times per week	44 (48.9)	16 (69.9)		
	Weekly	23 (25.6)	2 (8.7)		
	Every 2-3 weeks	8 (8.9)	2 (8.7)		
	Monthly	2 (2.2)	0 (0)		
	Less than monthly	2 (2.2)	1 (4.3)		
	No regular contacts	11 (12.2)	2 (8.7)		
No previous experience with VR		82 (91.1)	17 (94.4)	0.218 (1)	.64

a VR: virtual reality.

b Higher VR acceptance group included the older adults who participated in at least the VR intervention and the posttest.

c Lower VR acceptance group included the older adults who did not want to join the VR intervention at the beginning (control group) and motivation-related dropouts; one of the motivation-related dropouts was excluded due to an incomplete baseline interview.

### Stimuli and Equipment

**Figure 2.**
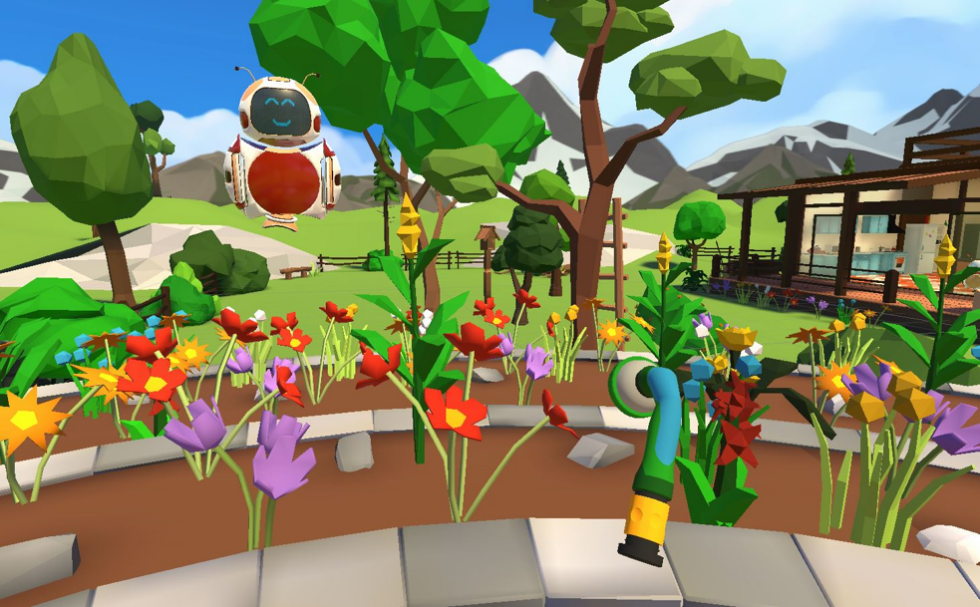
Virtual reality gardening task [27].

**Figure 3.**
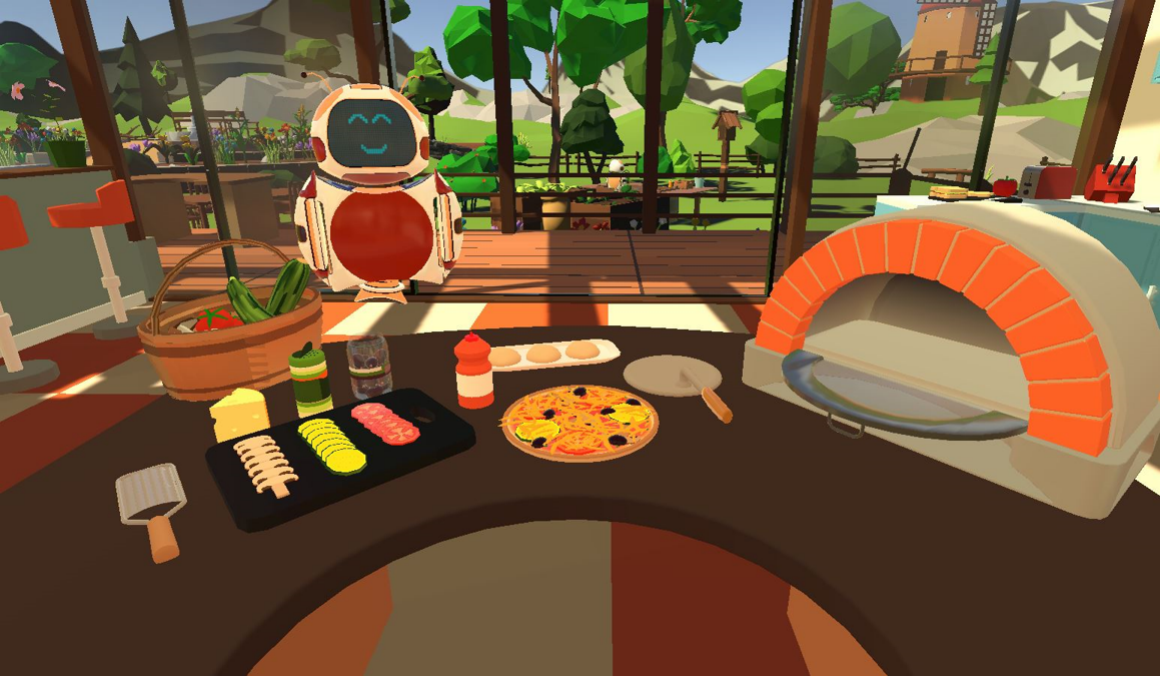
Virtual reality task in the kitchen: baking a pizza [27].

The VR intervention was done with a virtual vacation home scenario. During each VR session, the participants’ tasks were to complete 4‐5 activities such as gardening, crafting, and baking (see Figures 2 and 3 for examples). Participants started by crafting a chair, then arranged a garden around it, and finally, they could enjoy sitting on the chair while tending to the garden’s plants. In the virtual environment, participants worked on tasks individually. After removing their VR headsets, they had the opportunity to discuss their experiences with each other in a group setting. Every VR event lasted approximately 20‐30 minutes, depending on the tasks assigned for each session.

The Pico Neo 3 Pro VR headset and Pico Neo 3 controller were used. The resolution of the head-mounted display was 1832 × 1920 pixels per eye, with a refresh rate of 72 Hz and 6-degrees-of-freedom inside-out tracking.

### Data Collection

Psychosocial and cognitive capacities were assessed with the Mini-ICF-APP scale [46]. In this study, the reference context for capacity assessment was daily life in the nursing home. The Mini-ICF-APP covers the following capacity dimensions: (1) adherence to regulations, (2) planning and structuring of tasks, (3) flexibility, (4) competence and knowledge application, (5) ability to make decisions and judgments, (6) proactivity and spontaneous activities, (7) endurance, (8) self-assertiveness, (9) contact with others, (10) group integration, (11) intimate relationships, (12) self-care, and (13) mobility. Each dimension is rated on an 8-point scale (from 0 being “This is a strength of mine,” to 7 indicating “This is impossible for me”). The Mini-ICF-APP is a heterogeneous scale, covering different psychosocial capacities. Each item can be interpreted individually because all items reflect different capacities. If a mean score across the 13 items is calculated, this can be interpreted as a global capacity impairment level. The Mini-ICF-APP is a standard scale for measuring psychosocial capacities that has already been validated in different languages [46-48]. The interrater reliability in our study ranged from *r*=0.446 (untrained) to *r*=0.910 (trained).

The structured questionnaire addressing technology engagement asked for the frequency of engagement with 4 distinct technologies: televisions, smartphones, PCs or tablets, and other novel technologies. Responses were recorded on a 5-point Likert scale, spanning from 1=never/not at all to 5=several times every day/very much.

To assess the acceptance of VR technology, two questions were asked, one about the participant’s overall perspective regarding VR and another about the inclination to participate in a VR group activity again. Each item was rated on a scale from 1=not at all to 5=very good/very much.

The VR group activities and the questionnaires were conducted in the nursing home, integrated as a daily event for the participants. This is the basis for ecological validity of the data.

### Statistical Analysis Plan

Older adults with higher and lower acceptance of using a VR intervention were compared. Initially, the higher and lower acceptance groups were defined. Participants were categorized into four groups: (1) no interest in the VR intervention but willing to participate in the interview (lower VR acceptance), (2) dropped out due to motivational reasons (lower VR acceptance), (3) dropped out due to nonmotivational reasons, and (4) completed the VR intervention program and the postinterview (higher VR acceptance).

Participants who dropped out for nonmotivational reasons were excluded from the study. Categories 1 and 2 were allocated to the “lower VR acceptance” group, while category 4 represented the “higher VR acceptance” group.

Sociodemographic data, psychosocial capacities, and technological engagement of the older adults were collected from both groups to enable us to answer the research question and hypothesis 1. Additionally, data on technological engagement and attitudes toward VR technology and VR intervention as a group activity were collected from the posttests of the higher VR acceptance group to analyze changes in acceptance within this group to provide answers to hypotheses 2 and 3.

Data were analyzed with the statistical software SPSS (version 29; IBM Corp [49]). We conducted *t* tests and *χ*^2^ tests for group comparisons of sociodemographic factors. Psychosocial capacities and technological engagement between the higher and lower VR acceptance group were analyzed with a two-tailed *t* test. We used a *t* test for paired samples to compare the degree of technological engagement within the higher acceptance group at baseline and post interview.

### Ethical Considerations

This research was funded by the German Federal Ministry of Education and Research (BMBF), project number 16SV8561 VRalive. This research was approved by the ethics committee at Technische Universität Braunschweig (FV-2020‐18). The study was preregistered in Deutsches Register Klinischer Studien on December 11, 2020. Informed consent, confidentiality, and data protection agreements were obtained from older adults or their legal guardian under the supervision of a caregiver. There was no compensation for participation. All activities conducted in the nursing home strictly adhered to the prevailing nursing home COVID-19 prevention and treatment policy. The VR goggles were diligently disinfected after each use.

## Results

There were no statistically significant differences (all *P* values >.05) in sociodemographic characteristics between both groups (Table 2).

Concerning psychosocial capacities (Table 3), older adults with lower VR acceptance were more proactive and had more robust social relationships in comparison with the older adults in the higher acceptance group.

**Table 3.** Self-reported impairment in psychosocial capacities according to the Mini-ICF-APP scale (0=that is clearly a strength of mine, 7=I am not able at all).

	Higher virtual reality acceptance group (n=90)		Lower virtual reality acceptance group (n=23)		*t* test (*df*)	*P* value	Effect size (*d*)
	Rating, mean (SD)	Impaired, % (rating≥5)	Rating, mean (SD)	Impaired, % (rating≥5)			
Adjustment to rules and routines	2.59 (0.89)	2.2	2.30 (0.97)	4.3	1.35 (111)	.18	0.31
Planning and structuring tasks	3.14 (1.83)	21.1	3.17 (1.85)	26	–0.07 (111)	.95	–0.02
Flexibility and adaptability	2.40 (0.92)	2.2	2.35 (1.30)	4.3	0.18 (27.89)	.86	0.05
Competence and knowledge application	2.27 (1.23)	5.5	2.04 (1.33)	4.3	0.76 (111)	.45	0.18
Ability to make decisions and judgments	2.63 (1.19)	8.9	2.39 (0.89)	0	0.91 (111)	.37	0.21
Proactivity and spontaneous activities	2.43 (1.20)	5.5	1.78 (1.44)	4.3	2.22 (111)	.03	0.52
Resilience and perseverance	2.56 (1.11)	3.3	2.57 (1.56)	12.9	–0.03 (111)	.97	–0.01
Self-assertiveness	2.60 (1.07)	5.5	2.13 (1.14)	4.3	1.86 (111)	.07	0.43
Contact with others	2.44 (1.43)	10	2.39 (1.44)	8.7	0.16 (111)	.87	0.04
Group integration	2.68 (1.36)	9.9	2.65 (1.77)	8.6	0.06 (28.99)	.95	0.02
Dyadic relationships	2.62 (1.63)	14.4	1.61 (1.56)	4.3	2.69 (111)	.008	0.63
Self-care	3.27 (1.71)	21.1	3.78 (2.43)	47.8	–0.96 (27.84)	.35	–0.27
Mobility	2.47 (1.49)	7.8	2.17 (1.30)	4.3	0.86 (111)	.39	0.20
Average score	2.62 (0.77)	5.5	2.41 (0.89)	4.3	1.14 (111)	.26	0.27

Older adults with low VR acceptance experience more self-care impairments and report a conservative stance toward adopting new technology (Tables3  4). In contrast, those with higher VR acceptance report being less active, with less meaningful social connections, but with higher self-care capacity and a more open attitude toward new technology (Tables3  4). There is no statistically significant difference in engagement with televisions (*P*=.62) and laptops (*P*=.25) between higher and lower acceptance groups (Table 4). In both groups, over 90% of participants use a television at least once per day. A contrast emerged in smartphone use, with 23.3% (21/90) of the higher acceptance group using it daily, while merely 4.3% (10/23) of the lower acceptance group do the same. These results support hypothesis 1, which postulated that the group exhibiting higher VR acceptance would report a higher level of technological engagement compared to the group with lower VR acceptance.

**Table 4.** Technological engagement of lower and higher VR^a^ acceptance groups at baseline.

	Higher VR acceptance (n=90)		Lower VR acceptance (n=23)		*t* test (*df*)	*P* value	Effect size (*d*)
	Rating, mean (SD)	Frequent use and high willingness, n (%) with rating≥4	Rating, mean (SD)	Frequent use and high willingness, n (%) with rating≥4			
Television^b^	4.46 (0.91)	84 (93.3)	4.35 (1.03)	21 (91.3)	0.49 (111)	.62	0.11
Smartphone^b^	1.91 (1.60)	21 (23.3)	1.17 (0.83)	1 (4.3)	3.04 (67.88)	.003	0.50
Laptop/PC^b^	1.68 (1.45)	16 (17.7)	1.35 (1.15)	2 (8.7)	1.16 (41.77)	.25	0.24
Other technologies^c^	3.31 (1.72)	50 (55.5)	1.70 (1.29)	3 (13)	4.97 (44.11)	<.001	0.98

a VR: virtual reality.

b Participants answered the question “How often do you use [technology]?” (1=never, 5=several times per day).

c Participants answered the question “Do you want to try other new technologies?” (1=not at all, 5=very much).

After their participation in the VR intervention (Tables5  6), older adults’ perspectives toward VR remained very positive and even increased, with many participants reporting looking forward to the next VR group event; therefore, hypothesis 2 (“After the VR group intervention, technological engagement among older adults will either be maintained or improved”) and hypothesis 3 (“After the VR group intervention, the acceptance of VR technology and willingness to participate will both align at a high level”) were confirmed as well.

**Table 5.** Technological engagement before and after a virtual reality intervention among higher virtual reality acceptance group participants (n=90).

	Baseline		Posttest		*t* test (*df*)	*P* value	Effect size (*d*)
	Rating, mean (SD)	Frequent use and high willingness, n (%) with rating≥4	Rating, mean (SD)	Frequent use and high willingness, n (%) with rating≥4			
Television^a^	4.46 (0.91)	84 (93.3)	4.49 (0.91)	84 (93.3)	–0.48 (89)	.63	–0.05
Smartphone^a^	1.91 (1.60)	21 (23.3)	1.77 (1.50)	16 (17.8)	1.37 (89)	.17	0.14
Laptop/PC^a^	1.68 (1.45)	16 (17.8)	1.60 (1.36)	14 (15.6)	1.26 (89)	.21	0.13
Other new technologies^b^	3.31 (1.72)	50 (55.6)	3.31 (1.67)	49 (54.4)	0.0 (89)	>.99	0.00

a Participants answered the question “How often do you use [technology]?” (1=never, 5=several times per day).

b Participants answered the question “Do you want to try other new technologies?” (1=not at all, 5=very much).

**Table 6.** Acceptance of VR^a^ following a VR intervention among higher VR acceptance group participants (n=88).

	Rating, mean (SD)	Positive responses, n (%) with rating≥4
How do you like VR technology in general? (1=not at all, 5=very much)	4.42 (1.07)	75(85.2)
Would you like to participate in another VR activity in the future? (1=not at all, 5=very much)	4.22 (1.39)	68(77.3)

a VR: virtual reality.

## Discussion

### Demographic Status

There were no differences in demographic status between the groups with higher and lower VR acceptance. This finding may seem to contradict previous research that suggested that factors such as age and education could predict the acceptance of new technology or VR [32,50]. The divergence in outcomes can be explained by the homogeneous sample in our study [51]. The participants in our study were selected based on their care requirements, creating a convergence of demographic and life status. The data from this naturalistic explorative study suggest that demographic factors have no significant impact on older adults’ VR group event participation.

### Psychosocial Capacities

Individuals within the lower acceptance group had a higher reported level of organizing daily activities and building dyadic relationships with trusted individuals. A potential reason for nonparticipation in VR activities among older adults in nursing homes is motivation-related. This supports the idea that new technology should align well with users’ life statuses and requests [52]. Among older adults, the adoption of technology for health purposes often aims to compensate for deficiencies [53]. Older adults with robust relationships may be better integrated with others and can meet their need for meaningful activities in their daily lives. Additionally, their capacity to organize activities may contribute to their lower intrinsic motivation for new activities, leading to a reduced perception of VR usefulness and subsequently decreased acceptance. The Senior Technology Acceptance Model [50] states that older adults with strong social relationships believed in the utility of technologies and were more inclined to use them compared to older adults with weaker social networks. Moreover, a study involving 31 participants conducted semistructured interviews, indicating that socially well-integrated older adults may not perceive a necessity to use socially assistive augmented reality systems [54]. The VR intervention in our project is oriented toward meaningful activities and entertainment, which may be less attractive for those older adults who are already engaged in social relationships within the nursing home. Therefore, the target group of VR intervention could be older adults with a lack of proactivity. These results refine the target group for VR group interventions in nursing homes, that is, older adults who are less active and have limited meaningful social networks. This aligns with the core objective of our VR study—to offer meaningful activities that enhance the activity level, capacities, and overall well-being of less active older adults.

### Technological Engagement and Attitude After VR Activities

Older adults with higher acceptance of VR technology also tend to demonstrate a stronger willingness to embrace new technological advancements. This indicates that, even in the context of VR as a group activity for older adults in nursing homes, an openness to new technology can be a predictor of higher acceptance.

Considering device usage, several factors warrant attention. First, television is already widely accepted throughout society, including among older adults in nursing homes [2]. Therefore, the acceptance of television was high in both groups.

Second, costs play a significant role [55]. Although the higher VR acceptance group showed greater engagement with smartphones, the actual usage rate remained minimal. One explanation may be that some older adults are facing financial constraints that hinder access to smartphones and PCs. Socioeconomic status is a factor that should not be neglected as a potential contributor to the acceptance of VR activities among older adults [32]. The Unified Theory of Acceptance and Use of Technology model by Venkatesh et al [56] expands on the TAM [30] by incorporating additional external factors such as social influence and facilitating conditions. Social influence refers to the extent to which an individual perceives that people they are close with believe he or she should adopt or utilize the new system. Facilitating conditions are characterized by the individual’s perception of the organizational and technical infrastructure available to support system usage. Social support is crucial in affording older adults the opportunity to access new technology and in facilitating their learning process, consequently bolstering their technological engagement [44,50]. If nursing homes could provide access to new technologies such as VR, this could potentially enhance technology engagement among older adults.

Third, the difference in engagement between laptops and smartphones may be attributed to perceptions of usability, ease of use, and cost considerations. Smartphones, with their convenient accessibility and added functionality (such as phone calls), are perceived as more useful among older adults in nursing homes. This aligns with one of the key concepts of the TAM: the perception of usefulness. Therefore, it is crucial to reiterate that to enhance the acceptance of VR group activities as meaningful experiences for nursing home residents, these activities should be tailored to meet the needs and preferences of older adults.

Furthermore, after the intervention, a positive attitude toward VR was evident in participants’ responses. This aligns with the idea that users often experience contentment with new technology once they use it [44,45], as well as with the Senior Technology Acceptance Model framework [50]. This suggests that more older adults, even those with lower VR acceptance, should be encouraged to give VR a chance. Such efforts could be facilitated by trusted individuals such as long-term social workers within nursing homes.

### Limitations, Potential Applications, and Future Research

There are several limitations to this study. First, there was a small group of older adults with lower technological acceptance. The unequal sample sizes of the two groups resulted from the naturalistic group formation. The problem with unequal group sizes in research studies is that this can introduce bias and affect the statistical validity of the results. Additionally, unequal group sizes may impact the power of statistical tests, potentially making it more challenging to detect true differences between groups if the sample sizes are not balanced.

Second, there could be selection bias during recruitment. The initial selection of older adults was done by the caregivers, and their choices could be influenced by their expectations about the older adults. Additionally, the older adults who chose to participate in the study already had a willingness to experience the VR activity. Therefore, the results may not represent the entire population of nursing home residents, particularly those who declined to participate. This limits the external validity of the study.

Third, the behavior and responses of the older adults could be influenced by the Hawthorne effect, leading individuals to alter their actions based on the perceived expectations of the researchers. Moreover, the older adults might have enjoyed talking to the interviewer and could have overrated their attitudes toward the VR activity.

Lastly, a methodologically robust randomized controlled trial should be conducted to make conclusions about intervention effects possible.

On the other hand, this study offers a perspective from the implementation of VR group activities in a real-life setting, thus boasting high ecological validity. It specifically focuses on the acceptance of defined VR applications for residents in nursing homes as meaningful activities. This enables a better understanding of the factors influencing attitudes toward VR group activities and provides an opportunity to address the needs of the target population.

This study provides several insights regarding future VR intervention acceptance among older adults in nursing homes and rehabilitation facilities. First, demographic status does not impact the acceptance of a VR group activity among older adults in nursing homes. Second, this study underscores the importance of targeting specific groups and acknowledging individual differences in characteristics and needs. This research suggests that the target population for VR group activities in nursing homes should be residents lacking proactive capacity and social relationships. Future studies should address the particular needs of this population. Moreover, the findings emphasize the need for enhanced social support to boost technological engagement among older adults, thereby promoting greater acceptance of digital interventions to address their needs. This may involve nursing homes providing increased access to VR and other new technologies, or nursing staff fostering trust and offering encouragement to residents to participate in VR activities. Finally, future research in the domain of older adults’ technology acceptance could specify capacities such as activities of daily living or focus on specific subgroups within nursing homes.

### Conclusion

This study explored characteristics of older adults in nursing homes with varying levels of VR acceptance and their perceptions of VR after participation in a VR group activity. The study found no sociodemographic differences between older adults with higher or lower acceptance of VR activities; however, the findings suggest tailoring VR interventions to older adults who are less proactive. This is in line with the purpose of the VR group event in this study—to improve activity, psychosocial capacities, and well-being for inactive older adults in nursing homes. The data suggest that it can be fruitful to motivate older adults, including those with apparently lower technology acceptance, to use the opportunity to experience VR, potentially facilitated by the support of their trusted individuals.

## Abbreviations

TAM: Technology Acceptance Model
VR: virtual reality
